# Integrating novel therapeutic strategies in myeloproliferative neoplasms: Driving disease‐directed progress

**DOI:** 10.1111/bjh.70672

**Published:** 2026-07-09

**Authors:** Trung Q. Ngo, Maria Georgiou, Donal P. McLornan, Claire N. Harrison

**Affiliations:** ^1^ Department of Haematology University College London Hospitals NHS Foundation Trust London UK; ^2^ Department of Haematology Guy's and St. Thomas' NHS Foundation Trust London UK

**Keywords:** JAK inhibitor, myeloproliferative neoplasm, novel therapy, transplantation

## Abstract

Over the past decade, there has been a substantial increase in the diversity and number of therapeutic options for myeloproliferative neoplasms (MPNs). While many remain within the clinical trial arena, the clinician and patient community have seen more approvals reaching the clinic and a rethink on how best we should be approaching these disorders is required. In parallel, there needs to be a paradigm shift in end‐point considerations within MPN clinical trial design, with a growing emphasis on longer term disease stability and modification, molecular responses, histomorphological modification, reductions in complications (e.g. thrombotic complications or disease transformation) and improved survival alongside the conventional goals such as haematological responses and symptom and spleen improvements where relevant. Given the heterogeneity of these disorders, with often marked intrapatient variability, greater consideration must be given to optimised sequencing strategies and rational combination approaches and, importantly, an emphasis on patient‐specific trajectories. In this review, we appraise the contemporary therapeutic armamentarium across the MPN spectrum and explore how these advances may best facilitate increasing personalised approaches in an evolving and complex treatment landscape.

## INTRODUCTION

Myeloproliferative neoplasms (MPNs) represent a biologically and clinically diverse group of clonal haematopoietic stem cell disorders characterised by dysregulated myeloid proliferation, chronic inflammation and variable risks of thrombosis, disease progression and transformation to acute leukaemia.^1^ Although traditionally classified into three discrete disease entities—polycythaemia vera (PV), essential thrombocythaemia (ET) and myelofibrosis (MF)—MPNs exist along a continuum of disease biology and clinical manifestations.^2^ This heterogeneity is driven by differences in mutational profiles, pro‐inflammatory signalling and as yet unidentified, host factors, resulting in variability in symptom burden, impaired quality of life (QoL) and clinical outcomes that are only partially captured by using conventional diagnostic categories.

Traditionally, we measured benefit across MPN in terms of preventing thrombosis, haemorrhage and a theoretical reduction in risk of transformation rather than survival prolongation. More recently, symptom burden and QoL are recognised as central features, often independent of classical disease risk categorisation.^3^ Incorporation of patient‐reported outcome measures (PROMs) into clinical trials and practice has reinforced the need for therapeutic strategies that address these components versus focusing solely on thrombotic risk reduction. This adds further complexity into evaluating new therapies. The typically indolent natural history of MPN also poses challenges for trial design and understanding how best to introduce these therapies into routine care.^4^ Conventional end‐points, such as haematological and spleen/symptom responses, may not adequately capture meaningful long‐term benefit, particularly with respect to disease progression and survival. There remains an urgent need to validate surrogate end‐points, potentially molecular responses, histological changes or inflammatory biomarkers, to facilitate more efficient assessment of emerging therapies.

Questions have emerged recently regarding whether current disease classifications and treatment paradigms remain appropriate in the modern era.^5^ Molecularly focused frameworks, marrow histology or other facets may better reflect therapeutic vulnerability and prognosis than traditional more rigid classifications. Such approaches may also identify novel therapeutic goals, including achievement of minimal disease states and, in selected patients, treatment‐free remission—concepts remaining elusive in MPNs.^6^ Importantly, this does not necessarily imply uniform treatment intensification; emerging data suggest that some patients, such as those classified as ‘triple‐negative’ ET, may derive limited benefit from cytoreductive therapy.^7^

Finally, the expanding therapeutic landscape must be considered within ‘real‐world’ healthcare systems. In publicly funded economies such as the United Kingdom, integration of novel therapies will inevitably require careful evaluation of cost‐effectiveness, reimbursement pathways and long‐term value. Balancing clinical benefit, QoL improvement and economic sustainability will be essential as increasingly complex and potentially costly treatment strategies enter routine practice. The structure of this review reflects conventional disease pathways underpinning current clinical practice based upon the relevant British Society of Haematology guidelines.^8^, ^9^ We organise our discussion around considerations to the classical MPN entities, thoughts on accelerated phase and the role of allogeneic haematopoietic stem cell transplantation (allo‐HSCT) intending to provide a practical framework within which emerging therapies can be critically appraised and coherently integrated into current optimal standards of care.

## ESSENTIAL THROMBOCYTHAEMIA

Essential thrombocythaemia is perhaps the most heterogeneous of the classical MPN, where management strategies vary according to individualised risk factors and mutational profiling.^7^, ^10^ Furthermore, while the International Prognostic Score of thrombosis in ET (IPSET‐thrombosis) risk stratification is weighted by mutation type (higher risk with *JAK2* V617F mutation), for the most part treating clinicians still use age >60 years, sustained platelet count >1500 × 10^9^/L or disease‐related thrombosis or haemorrhage as indications for cytoreduction (‘high‐risk’ disease).^11^ For all other ‘low‐risk’ patients, the addition of cytoreductive treatment is generally considered on a ‘case‐by‐case’ basis for cardiovascular risk factors or symptom burdens. First‐line therapies are hydroxycarbamide (HC), pegylated interferon (IFN), with currently available second‐line therapies remaining anagrelide or busulfan.^10^ Unmet need for novel treatments in ET is most evident in those refractory or intolerant to standard first‐line therapies. Importantly, criteria for resistance or intolerance to HC exist but those for IFN do not, and resistance to each therapy might have differing biological and prognostic implications. Anagrelide and busulfan can be effective alternative treatments though each may have undesirable adverse effects. Second or later line options for ET patients include Janus Kinase (JAK) inhibitors (JAKi) and potentially other mutation‐specific agents, alternative formulations of interferon, bromodomain and extra‐terminal domain inhibitors (BETi), lysine‐specific demethylase‐1 inhibitors (LSD1i), cytidine triphosphate synthase 1 inhibitors (CTPS1i) and *CALR*‐directed immunotherapies (Table 1 and Figure 1).

**TABLE 1:** Novel therapies in essential thrombocythaemia.

Class	Drug	Trial(s)	End‐points	Clinico‐haematological response	Molecular response	Adverse events
Pegylated interferon/ Immunomodulator	Ropeginterferon alpha‐2b (Besremi)	**ROP‐ET** (**NCT06514807**, phase 3 prospective study. Resistance/ineligibility to other available treatments, never had interferon before) [6]	Assess if there is durable (≥3 months) haematological and clinical response after 12 months as per ELN criteria* **Secondary**: Thromboembolic/bleeding events, occurrence of disease progression, safety profile	48% of patients achieved durable response as per ELN criteria * after 12 months Major thrombotic events in 1.5% No haemorrhagic events, no disease progression	Reduction of 68.8% in *CALR* mutation and 64.3% in *JAK2* mutation at 12 months	Overall well tolerated ‐73% had AEs of which 68.9% were Grade 1 ‐Only 2.3% discontinued treatment due to toxicity
LSD1 inhibitor	Bomedemstat (MK‐3543)	**MK‐3543‐003 trial (NCT04254978**, phase 2 open label study) [7] **MK3543‐006 trial** (**NCT06079879**, phase 3, Bomedemstat vs. Best Available Therapy (BAT)) Currently Recruiting [8]	**MK‐3543‐003:** Assess safety and response, defined as platelets ≤400 × 10^9^/L without thromboses **MK3543‐006:** **Primary:** Safety and efficacy of Bomedemstat vs. BAT in refractory/intolerant to HC patients **Secondary:** Change of symptom score, rate of disease progression, percentage of thrombotic/haemorrhagic events	**MK‐3543‐003:** 79% achieved durable reduction in platelet count ≤400 × 10^9^/L after 24 weeks No thrombotic events **MK3543‐006:** No available data	No available data	**MK‐3543‐003:** ‐Overall well tolerated ‐Main AEs: dysgeusia (52%), fatigue (34%), constipation (32%), arthralgia (27%), thrombocytopenia (25%) **MK3543‐006:** No available data
mut*CALR* targeted immunotherapy	INCA033989 (immunoglobulin G1 monoclonal antibody)	**INCA033989** phase 1 trial (**INCA33989‐101, NCT05936359** enrolling outside the U.S. and **INCA33989‐102, NCT06034002** enrolling in the U.S.) [9]	**Primary:** Dose limiting toxicities Treatment emergent adverse events **Secondary:** Response to ELN criteria* Symptom improvement as per MPN‐SAF TSS score Changes in mut*CALR* allele burden pharmacokinetics	Complete haematological response(CHR) as per ELN criteria* in 68% of patients and durable response^a^ for at least 12 weeks in 44% Recovery of haematopoiesis in bone marrow biopsy	Reduction in mut*CALR* vaf in 94% of patients 31% of participants achieved >50% mut*CALR* reduction	Grade ≥3 anaemia and/or neutropenia in 9% of patients (concomitant use of hydroxyurea (hydroxycarbamide) prior or during event) No grade ≥3 thrombocytopenia Overall well‐tolerated
Anti‐mut*CALR*/anti‐CD3 bispecific antibody	JNJ‐88549968	**88549968MPN1001** phase 1/2 trial, **NCT06150157** Total duration 2 years [10]	Assess safety profile of JNJ‐88549968 in Part 1 (Dose escalation) Assess safety and efficacy of effective dose determined in previous cohort in part 2	No available data	No available data	No available data

* ELN criteria for haematological response: CHR defined as platelet count ≤400 × 10^9^/L and leucocytes <10 × 10^9^/L, partial haematological response (PHR) defined as platelet count ≤600 × 10^9^/L and leukocytes <10 × 10^9^/L (baseline platelet count >600 × 10^9^/L).

^a^ Durable response defined as maintaining for ≥12 weeks.

**FIGURE 1:**
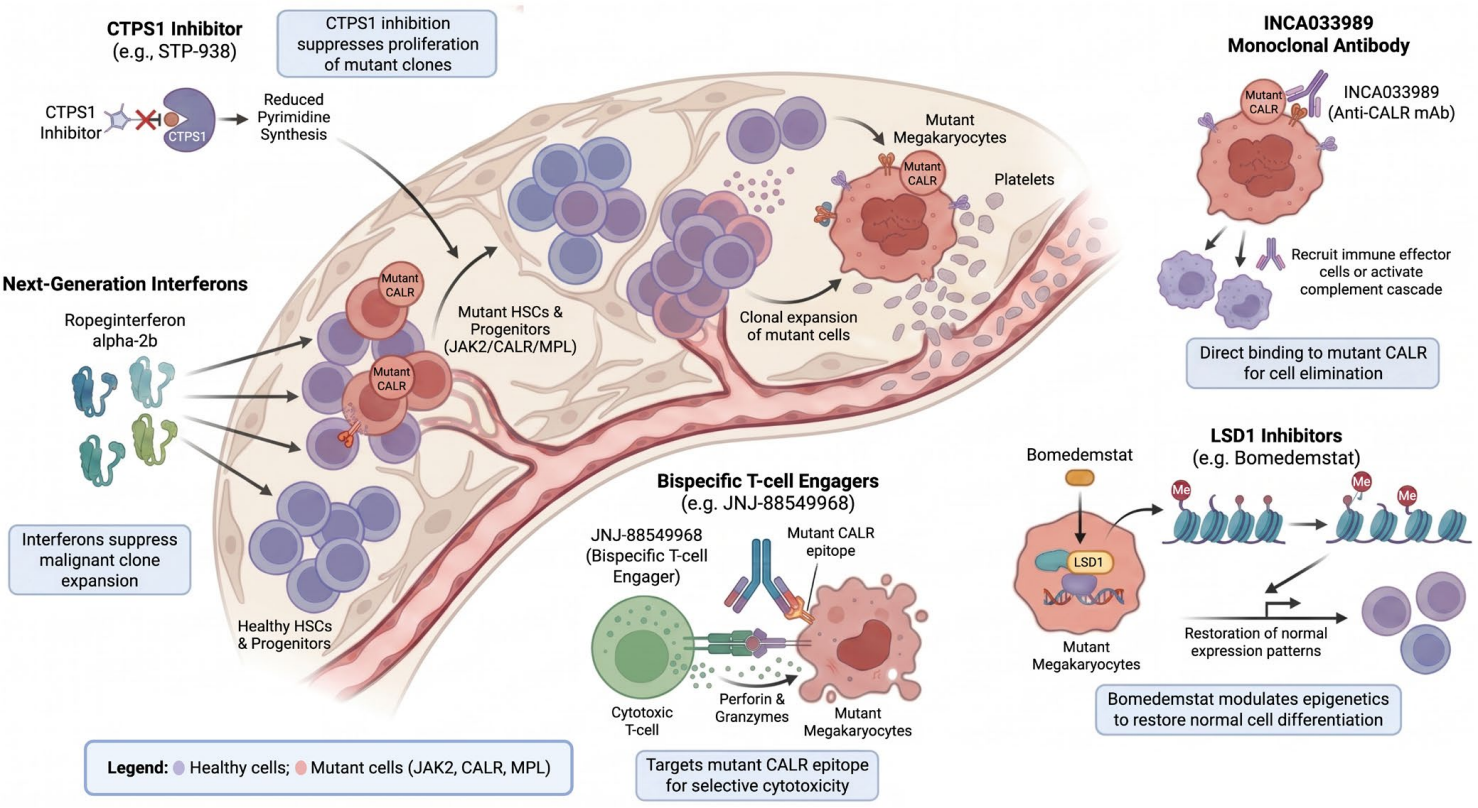
Mechanism of action of novel interferons and bispecific CALR antibody in essential thrombocythaemia.

Landmark clinical trials such as SURPASS‐ET (NCT04285086) and ROP‐ET (NCT05482971) highlighted the role of ropeginterferon alpha‐2b as an important alternative therapeutic in ET patients. SURPASS‐ET was an open‐label, randomised, phase 3 trial which included HC‐resistant/intolerant patients with white blood cell (WBC) count >10 × 10^9^/L, assigned to receive either ropeginterferon alpha‐2b or anagrelide. Ropeginterferon alpha‐2b demonstrated a significantly higher rate of durable haematological responses (43% vs. 6.0% in anagrelide arm) and superiority in controlling platelets, spleen size, symptoms and reducing haemorrhagic/thrombotic events compared to anagrelide. In addition, ropeginterferon alpha‐2b reduced the *JAK2* V617F variant allelic frequency (VAF) while anagrelide did not.^12^ Similar findings were showcased in the ROP‐ET trial where ropeginterferon alpha‐2b showed durable haematological response at 12 months and reductions in both *JAK2* and *CALR* VAF in patients who were intolerant/resistant or ineligible to other cytoreductive treatment (hydroxycarbamide, anagrelide, busulfan or pipobroman).^13^

Meaningful trial end‐points are particularly challenging for ET. The European LeukemiaNet (ELN) response criteria, published in 2013, reflect multiple disease facets; however, histological and molecular responses are often not prioritised in current trials.^14^ Data from trials with *CALR*‐directed immunotherapies (INCA033989) highlight that molecular and histological responses parallel cytoreduction and symptom improvement.^15^ Intriguingly, in this context, a ‘functional cure’ may not be as elusive as once thought, and hence, such therapies would need to be evaluated in an entirely different way to conventional treatments. The evidence for benefit using a formal cost/benefit analysis is also complex for ET given the long disease course. Novel therapies in ET are most likely to be approved in the second‐line setting where evidence of beneficial disease modification and safety might ultimately lead to front‐line utilisation and potentially extend to early treatment of selected classical ‘low‐risk’ ET.

## POLYCYTHAEMIA VERA

Polycythaemia vera is perhaps more ‘homogeneous’ than ET, almost all patients having a mutation in *JAK2*, and both ruxolitinib and ropeginterferon alpha‐2b have gained regulatory approval within the last 15 years.^16^ Though the latter is not available in the United Kingdom routinely and ruxolitinib was only approved after the UK MAJIC‐PV study (ISRCTN61925716) demonstrated significant benefit for event‐free survival (EFS).^17^ Data suggesting that modest molecular responses of ≥50% reduction *JAK2* V617F VAF correlates with survival and that complete haematological response (CHR) correlated with EFS supports further integration of these end‐points into current PV treatment algorithms and potentiates discussion of treating conventional ‘low‐risk‐PV’ more aggressively.^18^, ^19^ Below we discuss novel therapies in PV (summarised Table 2), while also being cognizant of the established role of HC and IFNs. Where hepcidin mimetics/targeted modulation, histone deacetylase (HDAC) inhibitors, LSD1 inhibitors and other novel agents will fit in the PV treatment paradigm requires careful consideration (Figure 2).

**TABLE 2:** Targeted and novel therapies in polycythaemia vera.

Class	Drug	Trial(s)	Endpoints	Clinico‐haematological response	Molecular response	Adverse events
*JAK2* Inhibitor	Ruxolitinib	**RESPONSE trial** (phase 3 trial, **NCT01243944** , ruxolitinib vs. standard therapy) **MAJIC PV trial** (phase 2 trial, **ISRCTN61925716**, ruxolitinib vs. Best available treatment (BAT)) **MITHRIDATE trial** (large phase 3 trial in UK/France, **NCT04116502**, ruxolitinib vs. BAT, Ongoing Recruitment)	**RESPONSE:** Haematocrit (Hct) control <45% until week 32 and spleen size reduction at least 35% at week 32 **MAJIC PV:** Complete response defined as: Hct < 45% without venesection for 3 months, platelets ≤ 400 × 10^9^/L, WBC ≤10 × 10^9^/L, normal spleen size **MITHRIDATE:** Primary end‐point: event‐free survival (EFS), progression‐free survival (PFS) Secondary: *JAK2* allele burden, symptom burden, spleen size	**RESPONSE:** At week 32: Primary end‐points achieved in 21% of ruxolitinib patients vs. 1% in standard treatment CHR in 24% of ruxolitinib patients vs. 9% on standard care **MAJIC PV:** Overall response 97% in ruxolitinib arm vs. BAT Higher PFS and thrombosis‐free survival **MITHRIDATE:** No available data	**Pooled analysis of RESPONSE:** *JAK2 V617F* variant allele frequency (vaf) reduced from 66.1% to 41.4% in 4 years **MAJIC PV**: Reduction of *JAK2V617F* vaf >50% in 56% of Ruxolitinib arm vs. 25% in BAT arm	**RESPONSE:** Grade 1/2 anaemia 8% in ruxolitinib arm vs. 8.2% in standard therapy, lower non‐haematological and thromboembolic events in ruxolitinib arm **MAJIC PV**: 29% grade 3/4 infections in ruxolitinib arm vs. 13% in BAT arm, squamous cell carcinoma in 11% of ruxolitinib arm
Hepcidin Mimetic	Rusfertide (PTG‐300)	**REVIVE trial** (phase 2, **NCT04057040** , rusfertide+ standard care vs. placebo+standard care) **VERIFY trial** (phase 3, **NCT05210790**, rusfertide+ standard care vs. placebo+standard care in part 1a, Rusfertide +standard care in part 1b)	**REVIVE**: *Primary end‐point*: Maintain haematocrit control (Hct <45%) without phlebotomy, *Secondary end‐point*: change in rate of phlebotomy **VERIFY:** *Primary end‐point*: Absence of phlebotomy eligibility, *Secondary end‐point*: improvement of PV symptoms	**REVIVE**: ‐Haematocrit control <45% without phlebotomy in 62% in rusfertide group vs. 14.8% in placebo. ‐Reduced phlebotomy rate in rusfertide group to <1.0/year **VERIFY:** ‐Sustained Hct control<45% through week 52. ‐Improvement of fatigue and MFSAF TSS7 symptom score in rusfertide vs. placebo ‐Rusfertide normalised ferritin and increased transferrin saturation	Not applicable	**REVIVE:** *Grade1/2*: All participants (fatigue, injection site reactions, prutitus, anaemia) *Grade 3*: 13% of participants No grade 4/5 **VERIFY:** Grade 1 in 86.3% in rusfertide group. Serious AEs in 3.4% of patients
Hepcidin Mimetic (*TMPRSS6* gene mRNA inhibitor)	Divesiran (SLN124)	**SANRECO trial** (phase 1, **NCT05499013**, divesiran vs. placebo) **SANRECO trial** (phase 2, divesiran vs. Placebo) Cytoreduction at stable doses allowed in both trials in both patient groups	**Phase 1:** Assess tolerability, safety and primary efficacy **Phase 2:** Haematocrit control <45% and phlebotomy independence between weeks 18 and 36	**Phase 1:** At 16 weeks, 50% of patients with Hct >50% required 7 phlebotomies. Patients with Hct <50% required no phlebotomy. Ferritin gradually increased, transferrin saturation reduced **Phase 2:** Completed enrolment. No published results	Not applicable	**Phase 1:** Well tolerated, 83% of SAE was grade 1 **Phase 2:** Ongoing
Hepcidin Mimetic (*TMPRSS6* gene modulator)	Sapablursen (formerly ISIS 702843)	**IMPRSSION trial** (Phase 2, **NCT05143957**, Cohort A—high dose, Cohort B—low dose. No placebo arm)	Efficacy, safety, tolerability, pharmacokinetics and pharmacodynamics of sapablursen in a 37‐week trial	Week 17–37: 53% in cohort A and 35% in cohort B achieved Hct control (<45%). MPN TSS score reduced by 32% in cohort A and 11% in cohort B. Transferrin Saturation persistently low Ferritin increased by week 37	Not applicable	Well tolerated 12.2% incidence of injection site reactions
Class I and II histone‐deacetylase inhibitor (HDACi)	Givinostat (ITF2357)	Phase 2 GIVINOSTAT trial (**NCT00928707**, givinostat 50 mg dose +MTD of HU group and givinostat 100 mg dose +MTD of HU patient group) [24] Long term phase 2 trial ( **NCT01761968** , givinostat at various doses with max dose 200 mg/d) [25] GIV‐IN‐PV phase 3 randomised trial (**NCT06093672**, Efficacy/safety of givinostat vs. HU): Ongoing Recruitment [26]	**NCT00928707**: Overall haematological response at Week 12. Secondary outcome: Change from baseline of the *JAK2V617F* Allele Burden **NCT01761968** : Long‐term efficacy and safety of givinostat monotherapy over a 4‐year observation period	**NCT00928707**: CHR or PR as per ELN criteria^a^ achieved in 55% of patients in 50 mg dose and in 50% in 100 mg dose of givinostat Reduced pruritus in 64% and 67% of patients respectively **NCT01761968** : 73% maintained PR for at least 4 years Absence of pruritus in 89% of patients Normalisation of spleen size in 56% of patients in 4 years	**NCT01761968:** Moderate decrease of 22% in *JAK2 V617F* vaf at 4 years	**NCT00928707:** Well tolerated, 1 patient on each arm had grade 3 AE **NCT01761968** : Well tolerated Five grade 3 AEs (thrombocytopenia, GI symptoms, asthenia). No grade 4/5 AEs
Lysine‐specific demethylase 1 (LSD 1) inhibitor	Bomedemstat	Phase 2 study **(NCT04262141)** [27]	Assess the haematological effects in PV patients who require control of their blood counts and are refractory to at least one cytoreductive treatment Secondary Endpoints: Treatment related toxicity, change in MPN SAF TSS score, change in spleen size, change in mutation allele burden	Reduction of Hct in more than 50% of patients to <45% by week 52 for at least 12 weeks without phlebotomy. 85% had WBC reduction to <10 × 10^9^/L in any time	No available data	Grade 3/4 AEs in 10% of patients (thrombocytopenia), No patients discontinued or died due to treatment‐related AEs

Abbreviations: CHR, complete haematological response; PR, partial response.

^a^ ELN criteria for complete overall haematological response: Hct (haematocrit) <45% without phlebotomy, and platelets ≤400 × 10^9^/L and leucocytes <10 × 10^9^/L and absence of splenomegaly, and no disease‐related systemic symptoms (microvascular disturbances, pruritus, headache) measured by the MPN‐SAF TSS score. Partial response: Hct < 45% without phlebotomy, or fulfilment of at least 3 of the other above‐mentioned criteria.

**FIGURE 2:**
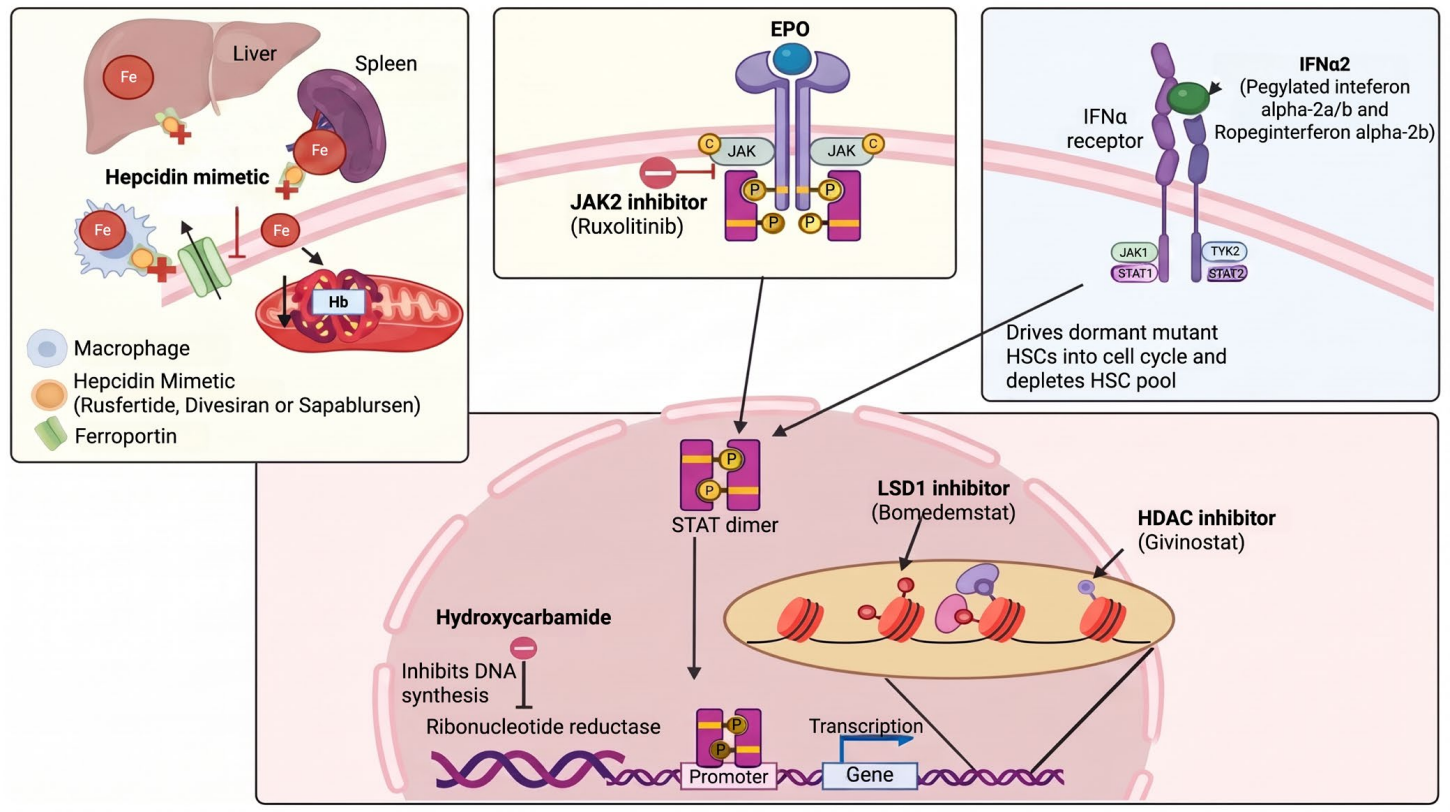
Mechanism of action of novel therapies in polycythaemia vera.

### JAKi in PV

Ruxolitinib is approved as a second‐line treatment for PV in patients with hydroxycarbamide intolerance/resistance. The phase 3 RESPONSE trial (NCT01243944) demonstrated that ruxolitinib was superior to standard therapy in achieving haematocrit control, spleen volume reduction (SVR) and symptom burden improvement in PV patients' resistance/intolerant to HC.^20^, ^21^ The UK MAJIC‐PV trial was an important milestone in the PV landscape, demonstrating wider benefits of ruxolitinib in achieving complete responses, higher molecular response rates and improved EFS.^17^ In our view, these findings highlight superiority in long‐term outcomes achieved with ruxolitinib, and thus, careful consideration should be given to use as a first‐line agent in PV, balanced against longer term safety concerns. The MITHRIDATE trial (NCT04116502) is a large phase 3 trial comparing ruxolitinib with either HC or IFN as first‐line therapy for high‐risk PV (high WCC >11 × 10^9^/L with one of: age >60 years, prior thrombosis or major haemorrhage, platelet count >1000 × 10^9^/L), currently open for recruitment in United Kingdom and France.^22^ The primary end‐point is EFS with secondary end‐points of reductions in symptom burden/spleen size and *JAK2* V617F VAF. It is an ambitious study but if it shows superior outcomes in the ruxolitinib cohort, it could significantly change the landscape of first‐line PV therapy.^22^

### Role of hepcidin mimetics

Hepcidin is a key regulator of iron metabolism, binding ferroportin to reduce iron absorption and availability for erythropoiesis. Rusfertide (PTG‐300) is a hepcidin mimetic first evaluated in phase 2 studies including REVIVE (NCT04057040) and PACIFIC (NCT04767802), which showed promising results for patients who failed to achieve haematocrit (Hct) control with or without cytoreduction. The REVIVE trial was divided into a 28‐week open‐label dose‐finding phase, followed by a 3‐month double‐blind randomisation (rusfertide compared to placebo), and a 3‐year open‐label extension, where rusfertide was administered to all patients and investigators could make cytoreductive treatment dose adjustments. The average clinically effective dose was 40 mg weekly and rusfertide could provide durable and sustained control of Hct <45% either alone or when combined with cytoreduction in responding patients. Additionally, it normalised iron stores in as short as 4 weeks of therapy and improved patient's symptom burden with an acceptable toxicity profile (25.7% had grade 3 adverse events, no grade 4 events).^23^ The PACIFIC trial (single arm trial with rusfertide induction period of subcutaneous 40 mg biweekly followed by maintenance 40 mg weekly until Hct < 45%) corroborated the above efficacy results.^23^, ^24^ These findings have been supported by interim results from the phase 3 VERIFY trial (NCT05210790, part 1a and 1b of the study from baseline to week 52), in which 62% of frequently venesected patients achieved sustained responses, including those crossing over from placebo to rusfertide at week 32, alongside improvements in iron parameters and PROMs. Rusfertide was generally well tolerated with mostly grade 1 and 2 adverse events.^25^

Additional hepcidin pathway–targeted therapies are also emerging. Sapablursen (ISIS 702843), an antisense oligonucleotide targeting transmembrane serine protease 6 (TMPRSS6), demonstrated dose‐dependent Hct control without phlebotomy and reductions in symptom burden in the phase 2 IMPRSSION study (NCT05143957).^26^ Divesiran (SLN124), a TMPRSS6‐silencing small inhibitory RNA (siRNA) evaluated in the phase 1/2 SANRECO study (NCT05499013), reduced venesection requirements and was generally well tolerated, with further data awaited.^27^ Overall, hepcidin‐directed therapies show promise in controlling Hct and reducing phlebotomy dependence in PV, although their optimal positioning within treatment pathways remains to be defined. Avoiding venesections can improve QoL, minimise fluid shifts and associated symptoms, reduce the symptom burden linked to significant iron deficiency, and eliminate time spent in hospital, which can otherwise interfere with occupational, social, and family life. They could potentially be used in conventional ‘low‐risk’ PV in patients who are venesection‐dependent to maintain Hct <45%, in whom repeated venesections often lead to progressive iron deficiency and related symptoms. In high‐risk PV, we have several effective disease‐modifying treatments. The role of hepcidin mimetics here could be argued to be more limited to adjunctive alongside hydroxycarbamide or IFNs when maximum tolerated doses do not achieve phlebotomy independence. There is insufficient evidence to guide use alongside ruxolitinib at present.

## MYELOFIBROSIS

### Consideration to who and when to treat in lower risk MF?

There are an increasing number of prognostic scoring systems for both primary and post‐PV/ET MF, such as the Dynamic International Prognostic Scoring System (DIPSS) and Mutation‐Enhanced International Prognostic Scoring System Plus (MIPSS70+) alongside more novel machine learning prognostic tools.^28^, ^29^ While JAKi registration trials focused on demonstrating benefit in intermediate‐2 and high‐risk disease, it is well established that symptoms, QoL issues and splenomegaly also affect patients with low‐risk or intermediate‐1 disease.^30^, ^31^, ^32^, ^33^ JUMP, ROBUST and RUX‐MF studies included intermediate‐1 risk patients with 50%–57% achieving a ≥35% reduction in spleen volume (SVR35) after 24 weeks of ruxolitinib, comparing favourably to the pivotal COMFORT‐1 and COMFORT‐2 trials in more advanced‐risk disease, with lower rates of haematological toxicity and discontinuation.^34^, ^35^, ^36^, ^37^ However, there are concerns of potential toxicity without demonstrable benefit of delaying progression or prolonging overall survival (OS), as yet. The ReTHINK study (NCT02598297) attempted to assess progression‐free survival (PFS) in this population but failed adequate recruitment.^38^ Given advances, it may be time to reconsider a similar trial in the contemporary era. Ropeginterferon alpha‐2b has been assessed in pre‐fibrotic, low‐risk and intermediate‐1 risk MF in the P1101MF trial (NCT04988815), demonstrating promising results with spleen length reduction in 64%, TSS50 in 43% and reduction of driver mutant VAF in 53% of patients after 24 weeks.^39^ HOPE‐PMF (NCT06468033) is an ongoing phase 3 trial comparing ropeginterferon alpha‐2b with placebo in pre‐fibrotic, low‐risk and intermediate‐1 risk MF (DIPSS‐plus); assessment of the long‐term potential for disease modification and delayed rate of progression are key.^40^

### Current JAKi: Updates, challenges and unmet needs

Ruxolitinib was approved by the Food and Drug Administration (FDA) in 2011 and European Medicines Agency (EMA) in 2012 for the treatment of intermediate‐2/high‐risk MF.^41^ Both the pivotal phase 3 COMFORT‐1 (compared with placebo) and COMFORT‐2 (compared with best available therapy; BAT) trials demonstrated that ruxolitinib had superior SVR35 and ≥50% reduction in total symptom score (TSS50) rates after 24 weeks.^30^, ^31^ Post hoc analyses suggested that ruxolitinib resulted in a greater median OS (5.3 compared to 3.8 years) than placebo or BAT.^42^ Furthermore, patients who started ruxolitinib within 12 months of diagnosis tended to have a greater OS benefit than those who started later, which suggested a potential survival benefit of early ruxolitinib initiation.^43^ However, ruxolitinib treatment is commonly complicated by cytopenias, resulting in dose reductions with a median time on treatment of around 3 years.^44^ Real‐world data illustrate that cytopenias correlate with reduced achievement of symptoms and OS end‐points.^45^ Consequently, other JAKi have been designed to bypass haematological toxicities; aiming to target spleen and symptom burdens alongside improving anaemia, for example, momelotinib (JAK1/2 and activin A receptor type 1 (ACVR1) inhibitor), fedratinib or pacritinib (currently only approved by the FDA). Furthermore, the FDA recently approved an extended‐release formulation of ruxolitinib, taken once daily, in May 2026, based on results of a phase 1 clinical trial (NCT06555081), which demonstrated bioequivalence and a similar safety profile with the immediate‐release formulation.^46^ The placement of different JAKi is discussed in British Society of Haematology guidelines and other papers.^47^, ^48^

JAKi are the backbone of MF therapy but have limited ability to modify the disease, such as inducing sustained reductions in fibrosis or mutational burdens. This highlights the need to combine JAKi with other novel therapies (Figure 3; Table 3). The subsequent trials we present below have commonly assessed novel drugs as monotherapy followed by combinations in relapsed/refractory settings initially, then in front‐line settings. None of the first three combinations (ruxolitinib with navitoclax, pelabresib or selinexor) successfully outperformed ruxolitinib monotherapy for symptom control, highlighting potential issues with trial end‐points. However, as discussed below, the phase 3 portion of the SENTRY trial did show a potential promising OS benefit with the selinexor–ruxolitinib combination over ruxolitinib monotherapy.^67^ No other therapies are currently approved for MF but likely we will shortly be using combination in the first and subsequent line of therapy. Given the rapidly evolving therapeutic landscape in MF, this review includes selected data presented in conference abstracts and press releases to maintain a contemporary overview. However, long‐term follow‐up and further analyses from these studies are necessary to fully establish efficacy and safety outcomes.

**FIGURE 3:**
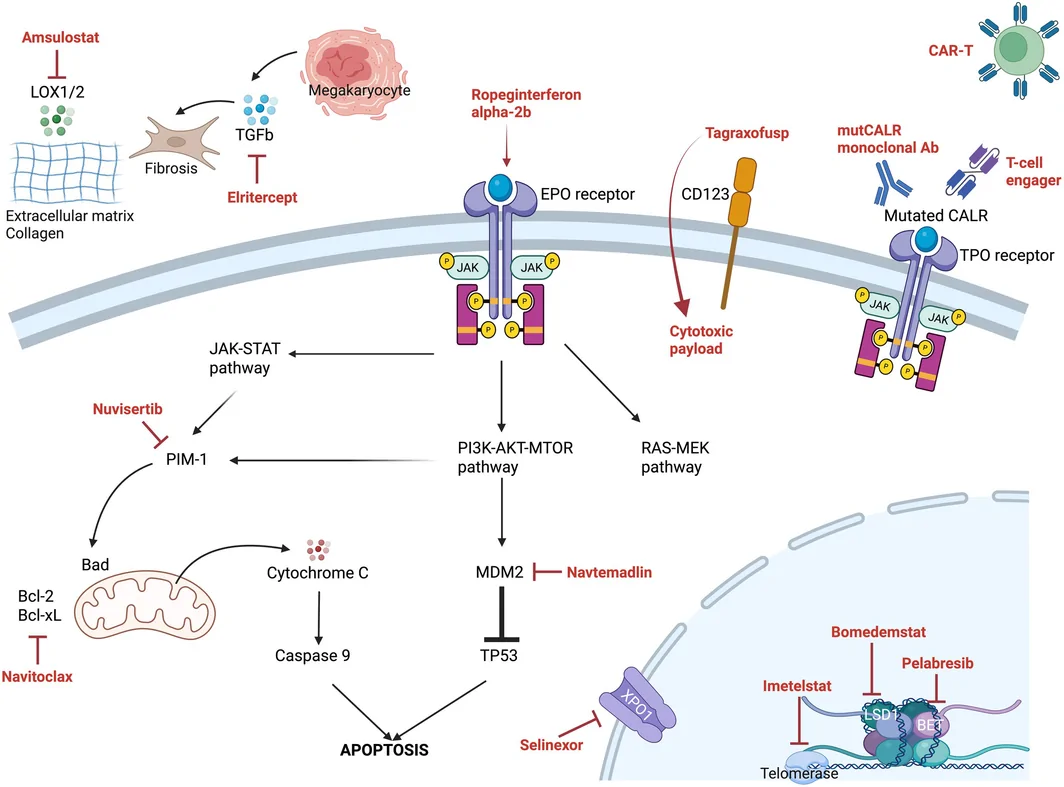
Mechanism of action of novel therapies in myelofibrosis.

**TABLE 3:** Novel therapies in myelofibrosis.

Class	Drug	Trial	SVR35 after 24 weeks	TSS50 after 24 weeks	Additional end‐points	Major limitations
Epigenetics						
BET inhibitor	Pelabresib‐RUX vs. PBO‐RUX	**MANIFEST‐2, publication** 49, 50 Phase 3, Frontline (NCT04603495)	66% vs. 35% **(*p* < 0.001)**	52% vs. 46% (NS)	Pelabresib‐RUX had greater: ↓cytokines & fibrosis; Signal towards ↓mutant VAF	OS benefit remains uncertain; symptom end‐point not met
LSD1 inhibitor	Bomedemstat‐RUX	**MK3543, conference abstract** 51, 52 Phase 2, Frontline and R/R (NCT05223920)	Frontline: 39% R/R: 7%	Frontline: 31% R/R: 26%	↓cytokines & fibrosis	Small sample size; limited long‐term efficacy data
Apoptosis and other novel targets						
MDM2 inhibitor	Navtemadlin Vs BAT	**BOREAS, conference abstract** ^53^ Phase 3, R/R (NCT03662126)	15% vs. 5% (*p* = 0.08)	24 vs. 12% (*p* = 0.05)	Navtemadlin had greater: ↓fibrosis & mutant VAF	Small sample size; early trial discontinuation
MDM2 inhibitor	RUX + add‐on Navtemadlin vs. PBO if suboptimal response	**POIESIS, data not available** Phase 3, Frontline (NCT06479135)	Not available	Not available	Not available	Not available
XPO1 inhibitor	Selinexor‐RUX vs. PBO‐RUX	**SENTRY phase 3, conference abstract** ^48^ Phase 3, Frontline (NCT04562389)	50% vs. 28% **(p < 0.001)**	Absolute TSS: −9.89 vs. −10.86 (NS)	Selinexor‐RUX had greater: Early signal of improved OS; SVR35 associated with OS advantage ↓mutant VAF	Symptom end‐point not met; thrombocytopenia and nausea may limit tolerability
PIM1 inhibitor	Nuvisertib (TP‐3654)	**TP3654, conference abstract** ^54^ Phase 1/2, R/R (NCT04176198)	23%	54%	↓cytokines & fibrosis	Small non‐randomised cohort, limited durability data
Bcl‐2 inhibitor	Navitoclax‐RUX vs. PBO‐RUX	**TRANSFORM‐1, conference abstract** ^55^ Phase 3, Frontline (NCT04472598)	63% vs. 32% **(*p* < 0.0001)**	39% vs. 42% (NS)	Mutant VAF and high‐risk molecular marker results not yet published	Symptom end‐point not met; thrombocytopenia related to BCL‐xL inhibition
Telomerase inhibitor	Imetelstat	**IMbark, publication** 56, 57 Phase 2, R/R (NCT02426086)	10%	32%	↓cytokines, fibrosis and mutant VAF	Modest spleen response; thrombocytopenia may limit tolerability
Telomerase inhibitor	Imetelstat	**IMpactMF, data not available** Phase 3, R/R (NCT04576156)	Not available	Not available	Not available	Not available
BM microenvironment						
TGF‐β inhibitor	Elritercept‐RUX	**RESTORE arm B, conference abstract** 58, 59 Phase 2, R/R (NCT05037760)	Not available	19%	↓transfusion burden in 60% and ↓mutant VAF in 17% of patients	Small sample size
Pan‐lysyl oxidase (LOX) inhibitor	Amsulostat	**PXS5505‐MF‐101, publication** ^60^ Phase 2, R/R (NCT04676529)	0%	15%	↓bone marrow collagen	Small sample size; spleen end‐point not met
CD123 inhibitor	Tagraxofusp	**STML‐401‐0314, publication** ^61^ Phase 1/2, Frontline or R/R (NCT02268253)	0% (13% at any time)	16% (40% at any time)		Toxicity profile and durability remain uncertain
Mutant *CALR*‐targeted therapies						
Monoclonal antibody	INCA033989 +/− RUX	**INCA033989‐101/102, conference abstract** ^62^ Phase 1, R/R Cohort A: Monotherapy Cohort B: With RUX (NCT05936359)	A: 30% B: 25%	Absolute TSS: A: −4.7 B: −12.3	↓cytokines, fibrosis and mutant VAF	Immature efficacy and safety follow‐up
Bispecific T‐cell engager	INCA035784	**INCA035784, data not available** Phase 1, R/R (NCT07008118)	Not available	Not available	Not available	Not available
Bispecific T‐cell engager	JNJ‐88549968 +/− RUX/MMB	**JNJ‐88549968, data not available** Phase 2, R/R (NCT06150157)	Not available	Not available	Not available	Not available
CAR‐T		NA				
Novel *JAK* inhibitors						
*JAK2* pseudokinase domain binder	INCB160058	**INCB160058‐101, data not available** ^63^ Phase 1, R/R (NCT06313593)	Not available	Not available	Not available	Not available
Type II specific *JAK2* inhibitor	AJX‐101	**AJX‐101, conference abstract** ^64^ Phase 1, R/R to Type I JAKi (NCT06343805)	65% by week 12	74% by cycle 1	↓mutant VAF	Immature efficacy and safety follow‐up
*JAK* and *ACVR1* inhibitor	Gecacitinib vs. hydroxyurea	**ZGJAK016, publication** ^65^ Phase 3, Frontline (NCT04617028)	65% vs. 27% **(*p* = 0.0002)**	62% vs. 50% (ns)	↓fibrosis and mutant VAF	Early crossover from hydroxyurea (hydroxycarbamide) to gecacitinib
*JAK2* and *FLT3* inhibitor	Flonoltinib	**FMF‐01, conference abstract** ^66^ Phase 1/2a, Frontline and R/R (NCT05153343)	Frontline: 83% R/R: 70%	60% by cycle 2	↓fibrosis	Immature efficacy and safety follow‐up

Abbreviations: BAT, best available therapy; MMB, momelotinib; NS, not statistically significant; PBO, placebo; R/R, relapsed/refractory; RUX, ruxolitinib; SVR35, splenic volume reduction ≥35% after 24 weeks; TSS%, total symptom score improvement ≥50% after 24 weeks.

### Defining relevant study end‐points

Across many studies, achievement of SVR35 and TSS50 have continued to be used as primary end‐points for novel therapies in MF due to rapid data being available for subsequent drug approvals, but have been criticised as reductions in splenomegaly may not correlate with underlying disease modification.^68^ Furthermore, recent combination trials were effective at reducing splenomegaly but failed to demonstrate additional improvements in symptoms, possibly due to a ‘ceiling effect’ where symptoms may already achieve maximum benefit with JAKi monotherapy; thus, the true benefits of combinatorial strategies are disregarded too early.^63^ This suggests a shift is needed to prioritise splenic volume reduction and novel end‐points over symptom improvement. The drive behind combinatorial studies is to augment responses and trials are increasingly assessing end‐points such as reductions in driver mutation VAF, amelioration of marrow fibrosis, improved marrow topography and downregulation of inflammatory cytokines and attempting to correlate if these impact survival outcomes.

### Novel JAKi

Novel JAKi in phase 1 trials are gecacitinib (JAK and ACVR1 inhibitor), flonoltinib (JAK2 and Fms‐like tyrosine kinase 3 [FLT3] inhibitor), AJX‐101 (type II specific JAK2 inhibitor) and INCB160058 (JAK2 pseudokinase domain binder).^64^, ^65^, ^66^, ^69^ Current FDA‐approved JAKi described above are type I, which target the active conformation; however, JAK2 activity persists via reactivation of downstream signalling.^70^ Type II inhibitors may overcome this limitation. An example is AJX‐101, which had superior efficacy, achieving reductions in driver mutation VAF in murine models. It is currently being investigated in a phase 1 study for PMF and post‐ET/PV MF patients who have failed a type I JAKi (NCT06343805); preliminary results presented at European Hematology Association Congress (EHA26) demonstrated a SVR35 of 65% by week 12 and TSS50 of 74% by the end of cycle 1.^65^

### Defining treatment failure and suboptimal response in MF

Identifying patients with a suboptimal response to therapy, and therefore at risk of treatment failure, remains heterogeneous in real‐world practice. A recent expert consensus statement defined refractory disease as ≤20% reduction in SVR and minimal symptom response (≤20% reduction in intermediate and highly symptomatic patients) after ≥3 months of ruxolitinib at maximally tolerated doses.^71^ Relapsed disease was defined as ≥25% increase in spleen volume within 1 month or ≥30% increase in TSS within 3 months of an initial response. A prognostic model, known as RR6, has shown that suboptimal splenomegaly responses, ongoing transfusion dependence and poor ruxolitinib tolerability in the first 6 months of therapy can predict OS; however, it was less reliable in lower risk patients.^72^ A subsequent intermediate‐1 specific RR6 (iRR6) model was developed to provide a functional risk assessment for patients with intermediate‐1 risk disease.^73^ The phase 3 POIESIS study (NCT06479135) has implemented a less conservative definition of suboptimal response (SVR <35% and TSS <50% after 24 weeks) to assess the benefit of ‘add on’ mouse double minute 2 (MDM2) inhibitor navetamadlin.^74^

### Novel therapies

#### Epigenetics

Examples of epigenetic modifiers in MF include BET inhibitors (e.g. pelabresib) and LSD1 inhibitors (e.g. bomedemstat). BETi target epigenetic proteins, thereby downregulating nuclear factor kappa‐light‐chain‐enhancer of activated B cell activity and cellular proliferation.^49^ Pelabresib, an oral BETi, is evaluated in MANIFEST‐2 (NCT04603495), an ongoing phase 3, randomised, double‐blind trial comparing pelabresib–ruxolitinib with placebo–ruxolitinib in JAKi‐naïve patients.^75^ Although the primary end‐point of SVR35 was met (65.9% vs. 35.2%), there were no statistically significant differences in the secondary end‐points of absolute TSS change (−15.99 vs. −14.05) or rate of TSS50 (52.3% vs. 46.3%) after 24 weeks. However, similarities in TSS scores may be due to a ‘ceiling effect’ with a similar average TSS in the general population.^50^ These results were durable at the week 96 analysis.^76^ Furthermore, there was a greater reduction of pro‐inflammatory cytokines and marrow fibrosis, and a signal towards greater reduction in the driver mutant VAF. There was also a safety signal with transformation to accelerated/blast phase, but this was not seen with longer follow‐up. MANIFEST‐3 is planned in a subgroup of patients with baseline TSS score ≥15 to elucidate differences between pelabresib–ruxolitinib and placebo–ruxolitinib in highly symptomatic patients and further assess safety.

LSD1 results in demethylation of acetylated histones and inhibition of p53 methylation.^51^ NCT03136185 and NCT05569538 are both ongoing open‐label phase 2 studies assessing the efficacy of bomedemstat monotherapy (oral LSD1 inhibitor) and bomedemstat–ruxolitinib respectively.^52^, ^54^ Both demonstrated bomedemstat‐mediated symptom burden improvement with a favourable safety profile; however, combination therapy demonstrated more robust results. Combination therapy achieved SVR35 and TSS50 after 24 weeks in 39% and 31%, respectively, in frontline, and 7% and 26%, respectively, in the relapsed cohort. Main adverse events were diarrhoea and dysgeusia; however, there was no dose‐limiting toxicity. A phase 3 trial is required to compare the efficacy and toxicity of the bomedemstat–ruxolitinib combination with ruxolitinib monotherapy.

#### Apoptotic pathways and other novel targets

Novel therapies which induce apoptosis include MDM2 inhibitors (e.g. navtemadlin), and other novel therapies include export protein exportin 1 (XPO1) inhibitors (e.g. selinexor), proviral integration site for Moloney murine leukemia virus 1 (PIM1) kinase inhibitors (e.g. nuvisertib), B‐cell lymphoma‐extra large (Bcl‐2) inhibitors (e.g. navitoclax) and telomerase inhibitors (e.g. imetelstat).^53^, ^55^, ^77^, ^78^, ^79^ MDM2 is upregulated in MF via constitutive activation of the phosphoinositide 3‐kinase (PI3K)/Protein Kinase B (PKB)/mechanistic target of rapamycin (mTOR) signalling pathway and functions as a negative regulator of p53.^80^ Navtemadlin, an oral MDM2 inhibitor, was assessed in the BOREAS phase 3 trial (NCT03662126), comparing navtemadlin with BAT in relapsed/refractory MF.^55^ Despite initially promising results at week 24 with a trend towards higher rates of SVR35 (15% vs. 5%) and TSS50 reduction (24% vs. 12%) compared to BAT, neither was statistically significant. The study was ultimately underpowered, and early trial discontinuation ensued to focus on the phase 3 POIESIS trial (NCT06479135). There was also an increased reduction of mutant VAFs and marrow fibrosis with navtemadlin, suggesting wider potential. Toxicity included cytopenias and gastrointestinal effects, although anaemia and thrombocytopenia remained stable across the duration of treatment. The POIESIS trial (as above) is currently ongoing.^74^

Selinexor blocks the nuclear XPO1, which traps tumour suppressor proteins within the nucleus, resulting in apoptosis.^81^ A phase 1 dose escalation/expansion study (XPORT‐MF‐034; NCT04562389) demonstrated that the front‐line combination of selinexor–ruxolitinib in MF achieved SVR35 in 79% and TSS50 in 58% of patients after 24 weeks.^82^ There was a rapid reduction in driver mutation VAF, suggesting a potential role in disease modification. A phase 3 randomised study (SENTRY, NCT04562389) of selinexor–ruxolitinib compared with placebo–ruxolitinib recently reported top line results in March 2026; with a rapid and near doubling of patients achieving SVR35 by week 24, a higher proportion of patients achieving ≥20% reduction in driver mutation VAF and potential early signals of improved OS (hazard ratio 0.43 with 95% confidence intervals 0.19–1.00, *p* = 0.0222).^67^ Post hoc analyses at 12 and 24 weeks also suggested that improvement in SVR35 was potentially associated with prolonged OS. Although the combination arm lacked superiority in the symptom end‐point, both arms achieved similar symptom improvement compared to baseline, suggesting a ‘ceiling effect’. Further follow‐up is required. SENTRY‐2 (NCT05980806) is a further study assessing selinexor as front‐line backbone therapy with the addition of ruxolitinib or momelotinib if the initial response is deemed suboptimal.^83^

PIM1 kinase is a downstream target of the JAK‐STAT and PI3K/PKB/mTOR pathways.^84^ Nuvisertib, a selective PIM1 kinase inhibitor, has been shown to reduce splenomegaly and marrow fibrosis with limited thrombocytopenic toxicity in murine MF models.^85^ An ongoing phase 1/2 study (NCT04176198) of nuvisertib monotherapy in patients with relapsed/refractory MF has yielded promising preliminary results with SVR35 and TSS50 achieved in 23% and 54% of patients respectively.^77^ Mean haemoglobin and platelet counts were relatively stable, and only 15% of patients demonstrated grade 3 thrombocytopenia. This study has now been expanded to include the addition of ruxolitinib (arm 2) or momelotinib (arm 3) to nuvisertib; preliminary results are awaited with interest.^86^

BCL‐2 and BCL‐X_L_ are anti‐apoptotic proteins which act on mitochondria to inhibit apoptotic function.^87^ Navitoclax is an oral pan‐BCL‐2/ BCL‐X_L_ inhibitor which showed initial promise in the phase 3 randomised TRANSFORM‐1 trial (NCT04472598) with navitoclax–ruxolitinib combination achieving superior SVR35 (63.2% vs. 31.5%) but not TSS50 after 24 weeks, compared to placebo–ruxolitinib in JAKi‐naïve patients.^78^ However, dose‐limiting toxicity (thrombocytopenia in 90% of patients and hepatotoxicity signals in some) resulted in cessation of further navitoclax development in MF.^88^ Recent work in MPN murine models has suggested that Bcl‐X_L_ inhibition potentiates interferon‐induced apoptosis in MPN stem cells; hence, sequenced therapy may be attractive as a future therapy but requires clinical trial evaluation of agents targeting this axis given concerns thrombocytopenia could represent a broader class adverse effect.^89^

Malignant cluster of differentiation (CD)34‐positive cells in MF have enhanced telomerase activity and shortened telomere length.^56^, ^90^ IMbark (NCT02426086), a phase 2 trial, evaluated imetelstat (telomerase inhibitor) monotherapy in patients with relapsed MF.^57^ At week 24, the 9.4 mg/kg dose achieved a SVR35 rate of 10.2% and TSS50 rate of 32.2%, marrow fibrosis improved in 41% and reduction in the driver mutation VAF in 42%. Median OS of 30 months seen in the imetelstat 9.4 mg/kg group compares favourably with real‐world data of an OS of 12 months in this historical cohort. A post hoc analysis demonstrated that imetelstat reduced Interleukin 8 (IL‐8) and tissue necrosis factor alpha levels in a dose‐dependent fashion, and OS was higher in patients with lower baseline IL‐8 levels, confirming imetelstat's potential role in disease modification.^91^ The phase 1b dose confirmation and expansion trial (IMproveMF; NCT05371964) of imetelstat–ruxolitinib combination is ongoing.^92^ The phase 3 open‐label randomised trial (IMpactMF; NCT04576156) of imetelstat versus BAT is unique because of the primary end‐point of OS. Both these trials will provide further enlightenment on the efficacy and toxicity of imetelstat.^58^

#### BM microenvironment

Therapies targeting the bone marrow microenvironment include transforming growth factor beta (TGF‐β) inhibitors (e.g. elritercept), pan‐lysyl oxidase (LOX) inhibitors (e.g. amsulostat) and CD123 inhibitors (e.g. tagraxofusp).^60^, ^61^, ^93^ Elritercept is a modified activin receptor (ActR) type IIA ligand trap which targets the TGF‐β family, thereby restoring bone marrow haematopoiesis.^59^ RESTORE (NCT05037760) is evaluating elritercept monotherapy and elritercept–ruxolitinib combination in MF patients with anaemia.^60^ Preliminary results show that a reduction in transfusion burden occurred in 60% of patients overall, albeit total numbers were small. Moreover, 18% and 15% of patients achieved SVR35 and TSS50 respectively. Updated data in the elritercept–ruxolitinib combination group showed 30% of patients with transfusion‐dependent anaemia achieved transfusion independence and 19% achieved TSS50 after 24 weeks.^94^ These results suggest that elritercept may hold promise in treating the disease/therapy‐related anaemia of MF patients on ruxolitinib. A phase 3 study is being launched.

Amsulostat is a LOX inhibitor which was investigated in a phase 1/2a dose and cohort expansion study as monotherapy for patients with MF who had prior JAKi therapy (NCT04676529).^61^ Sixty‐two per cent of patients achieved ≥20% reduction in TSS and 15% achieved TSS50. Notably, 42% of patients had an improvement in collagen fibrosis by ≥1 grade. Median haemoglobin and platelet levels were stable. An add‐on‐phase is currently underway, to assess efficacy and tolerability of combination treatment.^95^

CD123, an interleukin receptor overexpressed in myeloid malignancies, has been investigated as a window for therapeutic targeting in MF.^96^ Tagraxofusp is a first‐in‐class agent that targets CD123 to deliver a cytotoxic payload of diphtheria toxin. A phase 1/2 study (NCT02268253) of tagraxofusp in relapsed/refractory MF has shown 40% of patients achieving TSS50 and the median OS was 19.3 months, comparing favourably to historical averages.^93^ Capillary leak syndrome occurred in 11% of patients during the first cycle. Development of this agent in MF is still underway and there may be a role for it in post‐allo‐HSCT maintenance strategies.

#### Mutant *CALR* ‐targeted therapies

There has been a rapid expansion of calreticulin (*CALR)*‐directed immunotherapies in MPN, generating excitement in the patient and clinical community. INCA033989 is a monoclonal antibody that targets mutant *CALR* in the TPO receptor complex.^62^ NCT05936359 and NCT06034002 are ongoing phase 1 trials examining the safety and efficacy of INCA033989 as monotherapy and in combination with ruxolitinib in relapsed/refractory *CALR*‐mutated MF.^97^ Updated results presented at EHA26 demonstrated INCA033989 therapy was well tolerated overall, with improvements in anaemia in a proportion. SVR35 was achieved in 31% and 38% of patients, and TSS50 was 37% and 31%, after 24 weeks in the monotherapy and combination groups respectively. Of note, >90% of patients in both cohorts had a reduction in mutant *CALR* VAF.

Recent developments have investigated other ways to target *CALR*‐mutated MF, such as with T‐cell engager bispecific antibodies and chimeric antigen receptor T‐cell (CAR‐T) therapy.^98^ A first‐in‐class CAR‐T product has been shown to effectively eradicate *CALR*‐mutant human cell lines and tumour cells in mice models.^99^ INCA035784 (NCT07008118) and JNJ‐88549968 (NCT06150157) are both bispecific T‐cell engagers which are currently being investigated in phase 1 studies; results are pending.^100^, ^101^

## HAEMATOPOIETIC STEM CELL TRANSPLANTATION

### Patient population

Allo‐HSCT remains the only curative therapy for MF but has traditionally been reserved for patients up to the age of 70 with intermediate‐2 or high‐risk according to the DIPSS or MIPSS70+ prognostic scores.^102^ The MF Transplant Scoring System (MTSS) incorporates functional status and transplant/donor details to predict outcomes but was modelled from a relatively small cohort of patients.^103^ The European Society for Blood and Bone Marrow Transplantation (EBMT) and ELN recommend front‐line allograft for MIPSS70+ high‐risk or DIPSS intermediate‐2 or high‐risk patients deemed low or intermediate transplant risk according to MTSS.^104^ It should be noted that subsequent validation of the MTSS in a Spanish cohort of MF allo‐HSCT patients did not generate the same degree of specific outcomes.^105^ The EBMT has subsequently harnessed machine learning to develop a survival model which assigns outcome predictions and non‐relapse mortality (NRM) risks for patients with MF undergoing allo‐HSCT, including patient and disease‐specific information alongside conditioning and graft‐versus‐host disease (GVHD) prophylaxis strategies.^106^ It identifies a unique population of patients who are deemed high risk of poor outcomes from transplantation, providing a useful tool to identify the optimal transplant candidate. Conditioning intensity for MF needs to strike a balance between efficacy and toxicity. A large retrospective EBMT study of 2224 patients by McLornan et al. showed similar OS rates between myeloablative (MAC) and reduced intensity conditioning (RIC).^107^ However, the MAC cohort had higher GVHD‐free relapse‐free survival (GRFS) and a trend towards a lower relapse rate. Consequently, the recommendation is for RIC conditioning in MF but consideration of non‐TBI‐based MAC conditioning in younger fit patients. Use of the Transplant Conditioning index (TCI) has been applied to MF allo‐HSCT ‐ here transplant candidates classified in the TCI‐intermediate or high conditioning cohorts had a lower cumulative incidence of relapse (CIR) compared to those with low TCI scores but at the cost of higher NRM.^108^

### Peri‐transplant JAKi

Experience of JAKi being integrated into the pre‐, peri‐ and post‐allo‐HSCT period as maintenance is increasing, with some early promising results. A recent phase 3 study (NCT03427866) evaluated pretransplant ruxolitinib from 14 days pre‐allo‐HSCT to 12 months post‐allo‐HSCT, which demonstrated a promising 1‐year GRFS rate of 71% and 2‐year OS rate of 82%.^109^ This was confirmed by a subsequent real‐world study, which showed significantly improved 1‐year GRFS rates in patients who continued ruxolitinib until engraftment compared to those who ceased pre‐conditioning (43% vs. 15%, *p* = 0.03).^110^ We see future thinking trials in allo‐HSCT using a combination of JAKi and novel agents in the transplant treatment plan, especially as potential maintenance strategies to reduce the considerable relapse risks. How these agents may or may not affect the clearance kinetics of MRD post‐allo‐HSCT will be of extreme interest and the best place/sequence of these novel therapies in the transplant algorithm will be a future challenge.

## ACCELERATED AND BLAST PHASE MPN

### Novel therapies

MPN in accelerated phase (10%–19% blasts in peripheral blood or bone marrow) and blast phase (≥20% blasts in peripheral blood or bone marrow) have dismal prognosis with a median OS of less than 12 and 6 months, respectively, regardless of treatment.^111^, ^112^ A recent retrospective analysis by Patel et al. examined 140 patients in blast phase (BP) and 62 patients in accelerated phase (AP).^113^ There was no difference in OS between patients treated with first‐line intensive chemotherapy, hypomethylating agent +/− JAKi or hypomethylating agent + venetoclax. Allo‐HSCT was performed in 65 eligible patients, which extended the median OS to 3.1 years from the time of diagnosis. Significantly, patients who were in complete response had a similar median OS from the time of allo‐HSCT to those who were in partial remission or a morphological leukaemia‐free state (2.85 vs. 2.30 years). This was significantly better than a median survival of only 0.74 years for patients who had stable disease. In patients with circulating blasts pre‐transplant, Gagelmann et al. showed that an increase in circulating blasts from 10% to 20% at the time of transplantation is associated with reduced OS and a greater risk of relapse.^114^ This highlights an area of unmet need for novel therapies prior to transplantation. Current clinical trials are investigating the role of fedratinib and oral azacitidine (FAMy trial), ruxolitinib and decitabine, ruxolitinib and CPX‐351 and ruxolitinib and IDH1/2 inhibitor in AP/BP MPN.^115^, ^116^, ^117^ There is a plan for a UK wide platform study in accelerated and blast phase MPN, which is much needed.

### Prognostic scores to predict progression to accelerated or blast phase: An unmet need

Transformation of chronic phase MF to AP/BP represents a significant management challenge and thus requires early identification. Current prognostic scoring systems, such as DIPPS and MIPSS70+ predict OS but do not truly capture the unique patient cohort who progress to AP/BP.^118^ Dynamic predictive signatures are needed. The peripheral blast count has been shown to play an important role, with a recent study demonstrating that chronic phase MF with 4% circulating blasts have similar clinical and prognostic features as AP disease.^119^ This points to an area of unmet need to refine prognostic scores to predict this unique cohort of patients and permit early intervention.

## CONCLUSION

In conclusion, MPN represent a complex and evolving spectrum of disorders in which traditional disease classifications and end‐points are increasingly insufficient to capture the full breadth of disease biology, patient experience and therapeutic impact. Advances in molecular understanding and the expanding range of targeted and combination therapies are reshaping management across ET, PV and MF, with growing emphasis on symptom burden, quality of life and potential disease modification. However, significant challenges remain, including the need for more meaningful and validated surrogate end‐points, improved risk stratification, particularly for disease progression, and clearer definitions of treatment response and failure. The integration of novel agents into clinical practice must also balance efficacy, safety and cost‐effectiveness within real‐world healthcare systems. Ultimately, future progress will depend on more biologically informed treatment strategies, refined clinical trial design and personalised approaches that align therapeutic intensity with individual patient risk and disease trajectory.

## AUTHOR CONTRIBUTIONS

**Claire N. Harrison:** Conceptualization; writing – review and editing; supervision; writing – original draft. **Maria Georgiou:** Writing – original draft; writing – review and editing; conceptualization. **Trung Q. Ngo:** Writing – original draft; writing – review and editing; conceptualization. **Donal P. McLornan:** Conceptualization; writing – review and editing; supervision; writing – original draft.

## FUNDING INFORMATION

No funding was received for this review paper.

## CONFLICT OF INTEREST STATEMENT

C.N.H. has received research funding from BMS, Novartis, GSK and Incyte, and advisory and speaker honoraria from AOP, BMS, IMAGO, MSD, Incyte, Novartis, Galecto, Geron, GSK, Janssen, Karyopharm, Keros, Menarini Stemline, Calytrix, Sobi, Takeda, Syntara, Daimura and Alethiomics. The other authors declare no competing interests. To ensure a balanced narrative review, selection and discussion of studies were agreed upon by all authors, based on scientific and clinical relevance.

## Data Availability

Data sharing is not applicable to this article as no datasets were generated or analysed during the current study.
